# Establishment of a Novel Baculovirus–Silkworm Expression System

**DOI:** 10.3390/microorganisms10051013

**Published:** 2022-05-12

**Authors:** Junhong Wei, Youpeng Fan, Xiaoling Jing, Zhihui Fei, Chunfeng Li, Guoqing Pan, Jialing Bao, Zeyang Zhou

**Affiliations:** 1 State Key Laboratory of Silkworm Genome Biology, Southwest University, Chongqing 400715, China; weijunhong@swu.edu.cn (J.W.); fanyoupeng1994@163.com (Y.F.); xljing2022@126.com (X.J.); fzh07237991@163.com (Z.F.); licf@swu.edu.cn (C.L.); gqpan@swu.edu.cn (G.P.); zyzhou@swu.edu.cn (Z.Z.); 2 Chongqing Key Laboratory of Microsporidia Infection and Control, Southwest University, Chongqing 400715, China; 3 College of Life Sciences, Chongqing Normal University, Chongqing 401333, China

**Keywords:** baculovirus, BmNPV, polyhedron, UAS/GAL4, recombinant virus

## Abstract

The baculovirus vector expression system is a well-established tool for foreign protein production and gene delivery. In this study, we constructed a recombinant baculovirus vector system. The UAS promotor region and *Bombyx mori* nucleopolyhedrovirus (BmNPV) polyhedrin coding region were ligated into a pFastBac Dual vector to obtain a BmBac-UPS recombinant bacmid. The recombinant bacmid BmBac-Gal4 was generated by the same strategy which has a Gal4 coding region controlled by the IE2 promoter. BmBac-UPS and BmBac-IGal4 were co-infected into silkworm BmN cells to confirm the ability of the UAS/Gal4 system to form polyhedrons in *B. mori* cells. Furthermore, the recombinant viruses were tested for infection efficiency and the ability to generate polyhedra in transgenic *B. mori* cell line BmE. The results showed that recombinant viruses have the ability to form polyhedrons and gain raised pathogenicity when orally infected *B. mori* larvae and are applied as the preferred tool for foreign gene delivery and expression

## 1. Introduction

*Baculoviridae* are composed of a large group of insect viruses that infect lepidopteran, hymenopteran, and dipteran insects [1]. As a result, baculoviruses are widely used in biological control and as bioreactors [2,3]. The baculovirus expression system is a reliable tool for producing high-quality recombinant proteins. In addition, baculoviruses can also be used to transduce mammalian cells, which expands their usage in biomedical fields such as gene delivery and gene therapy [4].

*Bombyx mori* (*B. mori*) neucleopolyhedrovirus (BmNPV) is a typical baculovirus which can infect silkworms and cause great economic losses [5]. Yet, researchers have engineered BmNPV as a vector for foreign gene expression and protein production with the aid of insect host cells [6,7]. This baculovirus expression system is characterized by high expression efficiency, folding and modification of expression products, and high biological activity. However, there are still limitations associated with the BmNPV expression system. For example, foreign proteins are usually expressed at a later stage of infection, leading to insufficient protein modification, while the invasion ability of the virus drops soon after being introduced into host cell populations [8,9,10]. More importantly, the virus vector needs to be injected into the silkworm to initiate infection and protein production because the *polyhedrin* gene (*polh*) has been disrupted so that no occlusion bodies could be formed to infect silkworms via oral inoculation [11,12]. Thus, it is necessary to construct a novel recombinant virus expression system for better performance.

The UAS-GAL4 system leads to the synthesis of levels of fusion proteins that exceed those of endogenous proteins by several orders of magnitude [13,14]. It will be of great interest and importance to investigate whether components such as polyhedrin and UAS-GAL4 could be assembled together into the baculovirus expression system and promote foreign protein production at high levels. In fact, Chen et al. showed the assembly strategy is feasible. They cloned the *polh* gene under the control of the IE1 promoter of BmNPV into a transposon pigA3GFP vector and then transported it into BmN cells to successfully construct a transgenic BmN cell line [11].

Here, in this study, we constructed and characterized a novel recombinant baculoviruses expression system. The BmBac-UPS recombinant virus has a polyhedrin coding region under the regulation of the UAS promoter and the BmBac-IGal4 recombinant virus has a Gal4 coding region controlled by the IE2 promoter. The ability of the recombinant viruses to generate polyhedra in transgenic silkworm cells was investigated. 

## 2. Materials and Methods

### 2.1. Insect Rearing and Cell Lines

The *B. mori* strain Dazao was reared on an artificial diet (Nihonnosanko, Yokohama, Japan) and maintained at 25 °C under a photoperiod of 12 h light and 12 h dark.

*Bombyx mori* cell line BmE-SWU cells were maintained at 28 °C in Grace’s medium (Thermo Fisher Scientific, Waltham, MA, USA) supplemented with 10% (*v*/*v*) fetal bovine serum (FBS) (Thermo Fisher Scientific, Waltham, MA, USA) and 1%(*v*/*v*) penicillin-streptomycin (Thermo Fisher Scientific, Waltham, MA, USA). *Bombyx mori* cell line BmN-SWU cells were maintained at 28 °C in TC-100 medium (Thermo Fisher Scientific, Waltham, MA, USA) supplemented with 10% (*v*/*v*) fetal bovine serum (FBS) (Thermo Fisher Scientific, Waltham, MA, USA) and 1% (*v*/*v*) penicillin-streptomycin (Thermo Fisher Scientific, Waltham, MA, USA). 

### 2.2. Cells and Viruses Production

**Figure 1:**
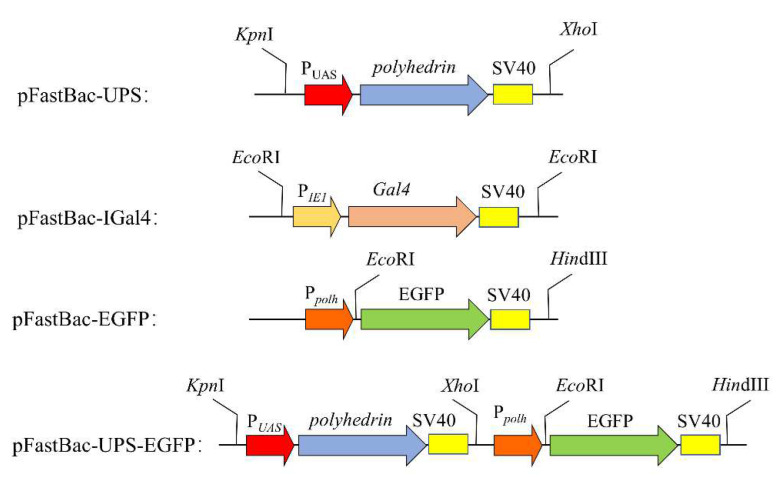
Construction strategies for recombinant Bacmids: pFastBac-UPS, pFastBac-IGal4, pFastBac-EGFP and pFastBac-UPS-EGFP.

The recombinant virus BmBac-UPS was constructed as follows: Polyhedrin (*polh*) driven by the UAS promoter cassette was synthesized and cloned into pUC57 to generate the pUC57-UASpol intermediate vector (Genewiz Inc., Suzhou, China). The fragment which contains both the UAS promotor region and polyhedrin coding region was then digested from pUC57-UASpol by KpnI/XhoI (New England Biolabs, Ipswich, MA, USA) and ligated into the pFastBac Dual vector to generate recombinant bacmid pFastBac-UPS. Next, we transformed pFastBac-UPS into BmDH10Bac™ *E. coli* (Thermo Fisher Scientific, Waltham, MA, USA) and transfected it into BmE cells according to the manufacturer’s instructions (Bac-to-Bac baculovirus expression system, Thermo Fisher Scientific, Waltham, MA, USA) to finally obtain a recombinant virus. The recombinant virus BmBac-Gal4 was constructed in the same way. Firstly, the Gal4 coding region driven by IE2 promoter was synthesized and cloned into pUC57 to generate Puc57-IGal4. The fragment which contains both the IE2 promotor region and the Gal4 coding region was digested from pUC57-IGal4 by EcoRI and ligated into the pFastBac Dual vector to generate recombinant plasmid pFastBac-IGal4. Next, we transformed the recombinant plasmid into BmDH10Bac™ *E. coli* and transfected it into BmE cells to obtain a recombinant virus. In order to obtain the recombinant virus pFastBac-UPS-EGFP, we firstly inserted the *egfp* gene under the ph promoter of pFastBac-UPS to generate the intermediate pFastBac-UPS-EGFP vector. The fragment containing both the Ppolh promotor and the EGFP coding region was digested and then ligated together with recombinant BmBac-UPS to obtain the recombinant bacmid. After transformation and transfecting in similar ways mentioned above, we obtain the recombinant virus pFastBac-UPS-egfp. Schematic illustrations of all vectors construction are shown in Figure 1.

### 2.3. Western Blotting

Western blotting was performed as previously described [15]. Cells were washed twice with PBS and lysed with protein lysis buffer (Beyotime, Shanghai, China) containing Phenylmethanesulfonyl Fluoride (Thermo Fisher Scientific, Waltham, MA, USA). After 30 min on ice, the lysates were incubated on ice for 30 min and then treated with 20% SDS-PAGE loading buffer (Beyotime, Shanghai, China) for 10 min at 100 °C. The samples were electrophoresed by SDS-PAGE and analyzed by immunoblotting using an anti-EGFP mouse monoclonal antibody (Roche Diagnostics, Mannheim, Germany) and Alexa Fluor 488–goat anti-mouse antibody (Sigma Aldrich, St, Louis, MO, USA).

### 2.4. Construction of Gal4 Transgenic BmE

The PiggyBac vector containing the coding sequence of Red Fluorescent Protein (DsRed), the neomycin resistance gene (*neo*) and the promoter IE2 driven Gal4 expression cassette was inserted into piggyBac vector to generate piggyBac[IE2-DsRed+IE2-Neo+IE2-Gal4]. The PiggyBac vector containing the coding sequence of Red Fluorescent Protein (DsRed), the *neo* and promoter 39K driven Gal4 expression cassette was used to generate piggyBac[IE2-DsRed+IE2-Neo+39K-Gal4]. The PiggyBac vector containing the Red Fluorescent Protein fused with the *neo* via P2A and Gal4 expression cassette was used to generate piggyBac[IE2-DsRed-P2A-Neo+39K-Gal4]. piggyBac[IE2-DsRed+IE2-Neo+IE2-Gal4], piggyBac[IE2-DsRed+IE2-Neo+39K-Gal4], and piggyBac[IE2-DsRed-P2A-Neo+39K-Gal4] were extracted from the DH5α cells with the EndoFree Mini Plasmid Kit II (TIANGEN, Beijing, China). BmN cells were transfected with 2 μg of this plasmid and a helper plasmid using X-tremeGENE HP DNA Transfection Reagent (Roche, Basel, Switzerland), and the culture medium was changed after 6 h. Three days later, the cells were cultured in Grace’s medium (Thermo Fisher Scientific, Waltham, MA, USA) containing G418 (200 μg/mL) (Merck, Darmstadt, Germany), and the culture medium was changed once every 4 d. The screening lasted for two months.

## 3. Results

### 3.1. UAS/Gal4 System Could Be Applied to Generate Occlusion Bodies in B. mori Cells

**Figure 2:**
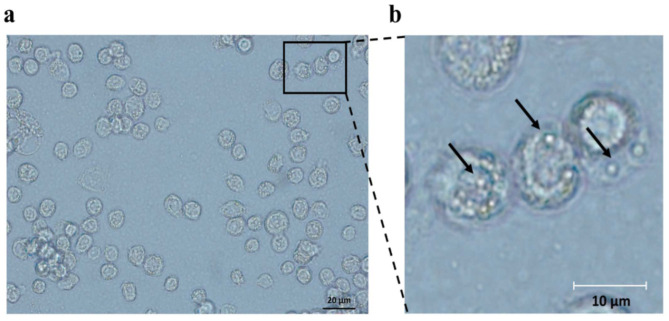
Detection of occlusion body in the BmN cells co-infected with Bm-UPS and Bm-Gal4. Virus-infected cells were visualized by microscopy at 72 h after infection under visible bright light. The scale bars indicate 20 μm (**a**) and 10 μm (**b**) respectively. Some of the inclusion bodies are indicated by arrowheads.

**Figure 3:**
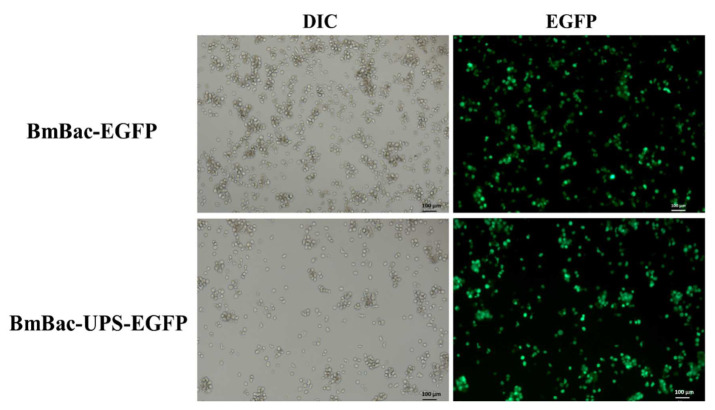
Detection of reporter gene expression in the BmN cells infected with BmBac-UPS-EGFPBmBac-UPS-EGFPBmBac-UPS-EGFP and BmBac-EGFP. Virus-infected cells were visualized by microscopy at 72 h after infection under differential interference contrast (DIC) and fluorescence (EGFP). The scale bars are 100 μm.

In order to confirm whether the UAS/Gal4 system could be applied for occlusion bodies’ formation in *B. mori* cells, we constructed recombinant baculoviruses BmBac-UPS which have *polh* under the regulation of the UAS promoter and BmBac-IGal4 which have a Gal4 coding region controlled by the IE2 promoter. BmBac-IGal4 and BmBac-UPS were used to co-infect BmN cells. The obtained results indicate the expression of polyhedrin in BmN cells and suggest they may package recombinant virus nucleocapsids to form occlusion bodies (Figure 2). To confirm the product’s expression ability of BmBac-UPS, *egfp* was inserted into BmBac-UPS as a reporter to generate BmBac-UPS-EGFP. were infected into BmN cells were infected with BmBac-UPS-EGFP and BmBac-EGFP, respectively. Expression was confirmed by fluorescence observation of EGFP (Figure 3), indicating that insertion of Gal4 expression cassette does not affect the expression of protein production in recombinant baculoviruses BmBac-UPS.

### 3.2. Infection Test of the Silkworm Larvae with the Occlusion Bodies

**Figure 4:**
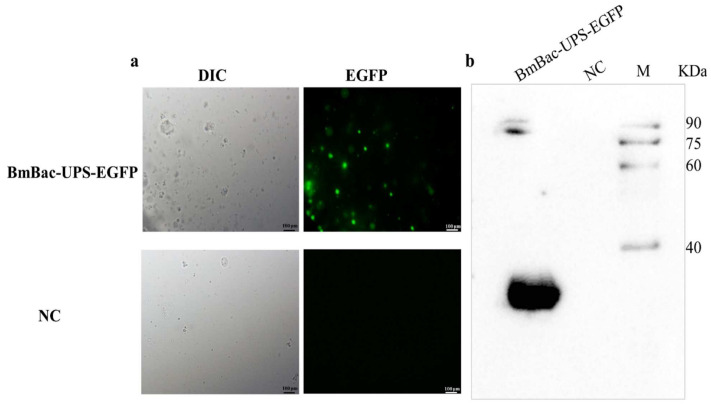
Infection test of the silkworm larvae with the occlusion bodies. (**a**) The blood of virus-infected larvae (BmBac-UPS-EGFP) and uninfected larvae (NC) was visualized by microscopy under visible bright light (DIC) and fluorescence (EGFP). The scale bars in the visible images are 100 μm. (**b**) Specificity of the HK antiserum. Proteins extracted from infected silkworm (BmBac-UPS-EGFP) and uninfected silkworm (NC) were subjected to Western blot using a polyclonal antibody against EGFP. M, Protein maker (Transgene, Shanghai, China).

To investigate the virulence of the packaged viruses, the *B. mori* larvae were orally infected with the occlusion bodies collected from BmN cells co-infected with Bm-IGal4 and BmBac-UPS-EGFP. The larvae exhibited a typical BmNPV infectivity phenotype, and the dead larvae were collected daily. Infection with recombinant baculoviruses was confirmed by fluorescence observation of EGFP in the blood (Figure 4a). Western blot was also performed and the result indicated expression of EGFP in the BmBac-UPS-EGFP-infected host (Figure 4b). 

### 3.3. Generation of the Transgenic BmE Cell Line

**Figure 5:**
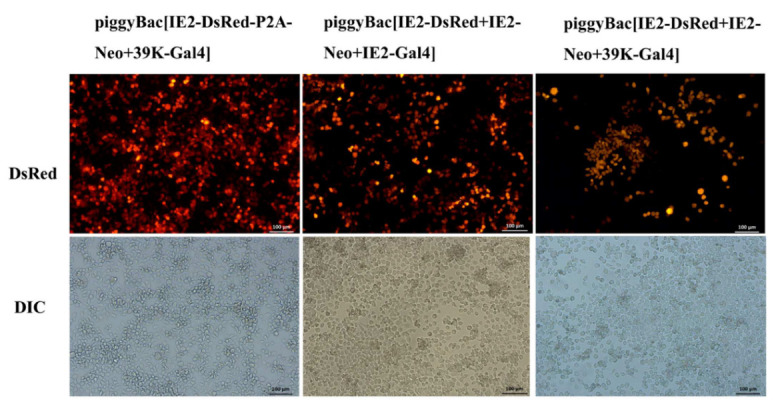
Observation of the transgenic BmE cells. For generating the transgenic BmN cells, the Gal4 expression vectors were mixed with the helper plasmid, respectively, and transfected into BmN cell by lipofection technique at 28 °C. The transgenic BmE cells were selected based on DsRed expression and G418 selection Panels: DsRed fluorescent field; DIC bright field. The scale bars in the visible images represent 100 μm.

For generating the stable transgenic BmE cell line, the BmE cells were selected based on DsRed expression and G418 selection. The observation of DsRed expression under the control of the IE2 promoter was confirmed by the observation under fluorescent microscopy. After several rounds of selection, the number of GFP-positive cells increased significantly (Figure 5). These results indicated that stable transgenic BmE cells were obtained. The results suggested that cell lines transfected by piggyBac[IE2-DsRed-P2A-Neo+39K-Gal4] showed a high rate of DsRed fluorescence, indicating they possess the potential to form an occlusion body. 

### 3.4. Generation of the Occlusion Bodies of Recombinant Baculoviruses in Transgenic Cells

**Figure 6:**
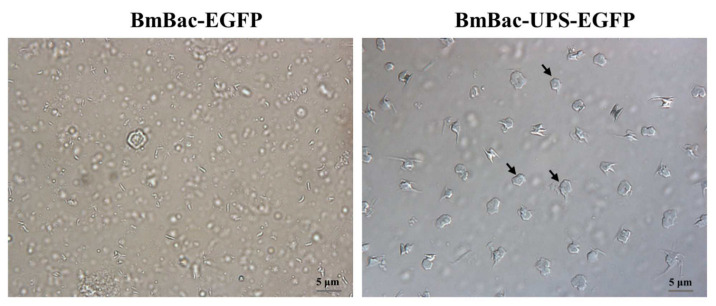
Detection of occlusion body in the transgenic BmN cells infected with BmBac-UPS-EGFP and BmBac-EGFP. Virus-infected cell lysates were visualized by microscopy at 120 h after infection under visible bright light. The scale bars in the visible images represent 5 μm. Some of the inclusion bodies are indicated by arrowheads.

In order to examine whether the recombinant virus could form occlusion bodies in the transgenic cells, the recombinant bacmids BmBac-EGFP and BmBac-UPS-EGFP were inoculated into the transgenic BmE cells, respectively. The occlusion bodies were observed in the medium of the transgenic BmE cells infected by BmBac-UPS-EGFP at 120 h post-infection (Figure 6). However, there were no occlusion bodies observed in a medium of the transgenic BmE cells infected by BmBcc-EGFP. The result suggests that expression products of the *polh* gene in the transgenic BmN cells can package the virus particles of BmBac-UPS-EGFP into occlusion bodies as expected.

## 4. Discussion

The baculovirus expression system mainly uses insect cell lines like Sf9 cells, while some researchers also focused on developing the robust high expression system using silkworms. As an important economic insect, silkworm *Bombyx mori* has numerous advantages in protein production such as low breeding cost and high efficiency of protein production. The mouse interleukin-3 production was about 500-fold higher in the hemolymph of infected silkworms than in the infected BmN cells [16]. In addition, the BmNPV-based baculovirus expression system has an advantage over the baculovirus AcMNPV because it has a narrower host range and will not grow in wild insect pests in the field [17]. However, currently, the BmNPV Baculovirus expression system needs intra-hemocoel injection of virus vectors to infect the silkworm, which greatly hinders efficiency in large-scale production of recombinant protein.

Large-scale production using *B. mori* larvae has been difficult so far because the inoculation of virus vectors requires a dorsal injection, which is relatively tedious and difficult [18]. In order to improve the infection rates and biosafety of baculoviruses as foreign gene delivery systems, we developed novel recombinant viruses in this study. We constructed a BmBac-UPS recombinant virus with a polyhedrin coding region under the regulation of the UAS promoter and a BmBac-IGal4 recombinant virus with a Gal4 coding region controlled by the IE2 promoter. The results indicated that the recombinant viruses have the ability to form polyhedrons, which means Polh is being produced, and infect silkworm larvae and transgenic cell lines.

This novel strategy of packaging a recombinant baculovirus is convenient and safer for the inoculation of *B. mori* larvae. Chen et al. have tried to inoculate the virus via oral inoculation [11]. However, in our study, we found that the growth of our constructed *polh*-expressing BmN cells seems abnormal, which may contribute to the constitutive expression of *polh* (unpublished data). In order to construct a virus-induced *polh* expression system, we applied UAS/Gal4 systems in this research because UAS/ GAL4 systems have been used in *B. mori* for targeted gene expression [19]. This system works effectively and safely in *B. mori* cells. 

In our experiment, we proved that the coexistence of both a UAS-driven *polh* expression cassette and an IE2-driven Gal4 expression cassette could start the expression of *polh* and package virus vectors into occlusion bodies in *B. mori* cells. The UAS-driven *polh* expression cassette in the recombinant virus did not affect the expression of the protein-producing cassette adjacent to it. The recombinant viruses BmBac-EGFP and BmBac-UPS-EGFP were unable to infect the *B. mori* larvae through oral inoculation. However, in our experiments, BmBac-UPS-EGFP could infect the *B. mori* larvae by oral inoculation through immobilization of the occlusion body in transgenic BmE cells with products of the *polh* gene. The virulence and protein expression ability of the recombinant virus were confirmed by fluorescence observation and Western blot of the infected larvae. The packaging of the recombinant baculovirus into the occlusion body will greatly facilitate oral inoculation of *B. mori* larvae regarding massive protein production, and will also significantly reduce bacterial infections caused by virus vector injection as well. This novel strategy of packaging recombinant baculoviruses will provide convenience and efficiency for the inoculation of *B. mori* larvae. For large-scale production of recombinant proteins by our system, occlusion bodies of virus vectors will be produced and collected in cultured transgenic cells. Then, purified occlusion bodies can be directly inoculated into silkworms by spraying them onto mulberry leaves. As the *polh* expression in the virus vector requires Gal4 protein in transgenic cells, no occlusion bodies will be formed during the protein production process in the silkworm because the *polh* will be silent in the host cells, which also greatly reduces the risk of transgene escape.

## Data Availability

Data will be available upon request.
